# Oncocytic pituicytoma in a patient with Cushing’s disease: a case report and narrative literature review

**DOI:** 10.3389/fendo.2025.1487120

**Published:** 2025-03-18

**Authors:** Jing Li, Shuaiming Chen, Huiwen Tan, Yerong Yu, Ying Tang, Bowen Cai, Jianwei Li

**Affiliations:** ^1^ Department of Endocrinology and Metabolism, West China Hospital of Sichuan University, Chengdu, China; ^2^ Department of Pathology, West China Hospital of Sichuan University, Chengdu, China; ^3^ Department of Neurosurgery, West China Hospital of Sichuan University, Chengdu, China

**Keywords:** Cushing’s disease, oncocytic pituicytoma, spindle cell oncocytoma, pituitary adenoma, posterior pituitary tumors

## Abstract

**Background:**

Posterior pituitary tumors (PPTs) are extremely rare, with fewer than 400 cases reported to date. In 2022, the World Health Organization (WHO) classified four types of tumors originating from the posterior pituitary: traditional pituicytoma, oncocytic pituicytoma, granular pituicytoma, and ependymal pituicytoma. To our knowledge, only one subject with coexistence of Cushing’s disease and oncocytic pituicytoma (spindle cell oncocytoma) has been reported, but the clinical features of this patient were not described in detail.

**Case presentation:**

We presented a case of a patient with Cushing’s syndrome and a pituitary mass. Transsphenoidal surgery was performed, and pathologic examination revealed two distinct tumors: a corticotroph adenoma with a diameter of less than 2 mm and a larger oncocytic pituicytoma. Post-surgery serum cortisol was 51 nmol/L, indicating complete remission. Corticotroph adenoma or corticotroph hyperplasia was identified after surgery in less than half of the subjects with Cushing’s disease and PPT.

**Conclusions:**

Our study indicates that Cushing’s disease in patients with PPT may be caused by the existence of collision lesions, with corticotroph adenoma or hyperplasia being difficult to detect due to their small dimensions.

## 1 Introduction

In 2022, the World Health Organization (WHO) redefined four types of tumors derived from the posterior pituitary, namely, traditional pituicytoma, oncocytic pituicytoma, granular pituicytoma, and ependymal pituicytoma (1). This update builds upon the WHO’s 2017 classification, which originally categorized these tumors as pituicytoma, granular cell tumor (GCT), spindle cell oncocytoma (SCO), and sellar ependymoma (2). However, this earlier classification was criticized for being nonspecific and potentially confusing, as it was not based on histogenesis. Consequently, in 2022, the 5th Edition of the WHO Classification of Endocrine and Neuroendocrine Tumors proposed that the oncocytic form, the granular cell form, and the ependymal type be classified as subtypes of pituicytoma. This change reflects a deeper understanding of the biomarkers associated with the pituicyte lineage, recognizing these tumors as subtypes of classical pituicytoma (1).

Posterior pituitary tumors (PPTs) are extremely rare, with fewer than 400 cases reported to date (3). These tumors are all low-grade nonneuroendocrine neoplasms, and their clinical symptoms and signs are primarily related to mass effect, which is similar to nonfunctioning pituitary macroadenomas (2). Studies have shown a relative high incidence of coexistence between PPT and adenohypophyseal hyperfunction, such as Cushing’s syndrome, acromegaly, and hyperprolactinemia (4). Most PPTs that coexist with adenohypophyseal hyperfunction are traditional pituicytoma and granular pituicytoma. To our knowledge, only one subject with coexistence of Cushing’s disease and SCO, which should be renamed as oncocytic pituicytoma in the new WHO classification, has been reported by far (5). However, the clinical features of this patient were not described in detail.

We presented a rare case of Cushing’s syndrome associated with a mass in the pituitary gland. Transsphenoidal surgery was performed, and pathologic examination revealed two distinct tumors: a corticotroph adenoma with a diameter of less than 2 mm and a larger oncocytic pituicytoma.

## 2 Case presentation

Written informed consent was obtained from the patient for the publication of any potentially identifiable images or data included in this article. A 41-year-old woman presented to the endocrinology department of West China Hospital with a 4-month history of a round face and hirsutism. Physical examination revealed the presence of a moon face, excessive facial hair, a buffalo hump, thinning skin, and scattered acne on the back. She had regular menstruation, and no previous history of hypertension or diabetes. Cushing’s syndrome was suspected, and initial workup included the following results: adrenocorticotropin hormone (ACTH), 44.39 ng/L; morning serum cortisol, 519.00 nmol/L; late-night serum cortisol, 375.00 nmol/L; and elevated 24-h urinary free cortisol, 833.4 μg/24 h. Serum cortisol at 8:00 a.m. the next day after 1 mg overnight dexamethasone suppression test (DST) was 549.00 nmol/L. Desmopressin acetate stimulation test (DDAVP) demonstrated a sevenfold increase of ACTH levels. Serum cortisol at 8:00 a.m. after standard large-dose DST (2 mg every 6 h consecutively for 2 days) was 189.00 nmol/L, and the suppression rate of 24-h urinary free cortisol was 69.8%. Therefore, Cushing’s disease was highly suspected.

**Figure 1:**
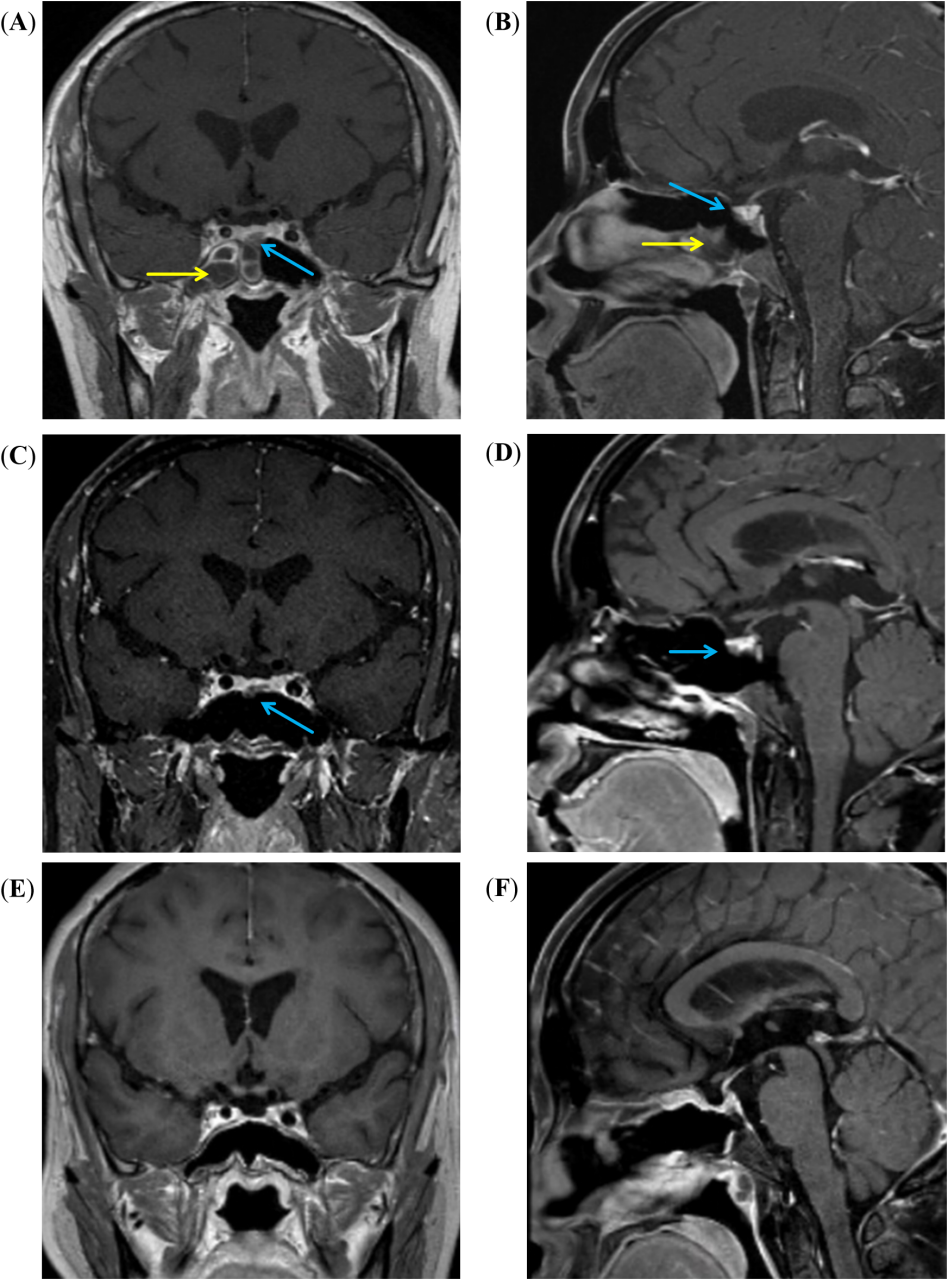
MRI images of the sellar region. **(A, B)** Coronal and sagittal T1-weighted enhanced MRI images show right sphenoid sinusitis (yellow arrow), and a low-signal intensity mass (blue arrow) is noted in the anterior-middle portion of the pituitary gland. **(C, D)** After 3 months of treatment with voriconazole, the right sphenoid sinusitis has resolved, but the mass (blue arrow) remains visible. **(E, F)** After the surgery, the pituitary tumor was resolved.

Contrast-enhanced magnetic resonance imaging (MRI) of the sellar region indicated right sphenoiditis and a slightly enhanced lesion with a diameter of 0.6 cm in the right lower lobe of the pituitary gland, and a microadenoma was suspected (**Figure 1**). Computed tomography (CT) scan of the nose confirmed the diagnosis of right sphenoiditis and also indicated maxillary sinusitis. Her sex hormones were within normal limits. Thyroid-stimulating hormone (TSH) was 1.56 mU/L (reference range: 0.27–4.2 mU/L), and free thyroxine (FT4) was 11.60 pmol/L (reference range: 12.0–22.0 pmol/L). Additional tests were also performed to screen for potential complications or comorbidities of Cushing’s disease. Oral glucose tolerance test showed impaired glucose tolerance and hyperinsulinemia, with a 2-h blood glucose level of 9.74 mmol/L and insulin at 123.0 μU/mL. Bone mass was normal, as shown by dual-energy x-ray absorptiometry (DXA), with *Z*-scores of −0.8 in the lumbar, −0.2 in the femoral neck, and −0.6 in the total hip. No spinal compression fractures were detected by x-rays of the spine. Additionally, she was diagnosed with hypertension, with a mean blood pressure of 133/93 mmHg, as measured by 24-h ambulatory blood pressure monitoring.

**Figure 2:**
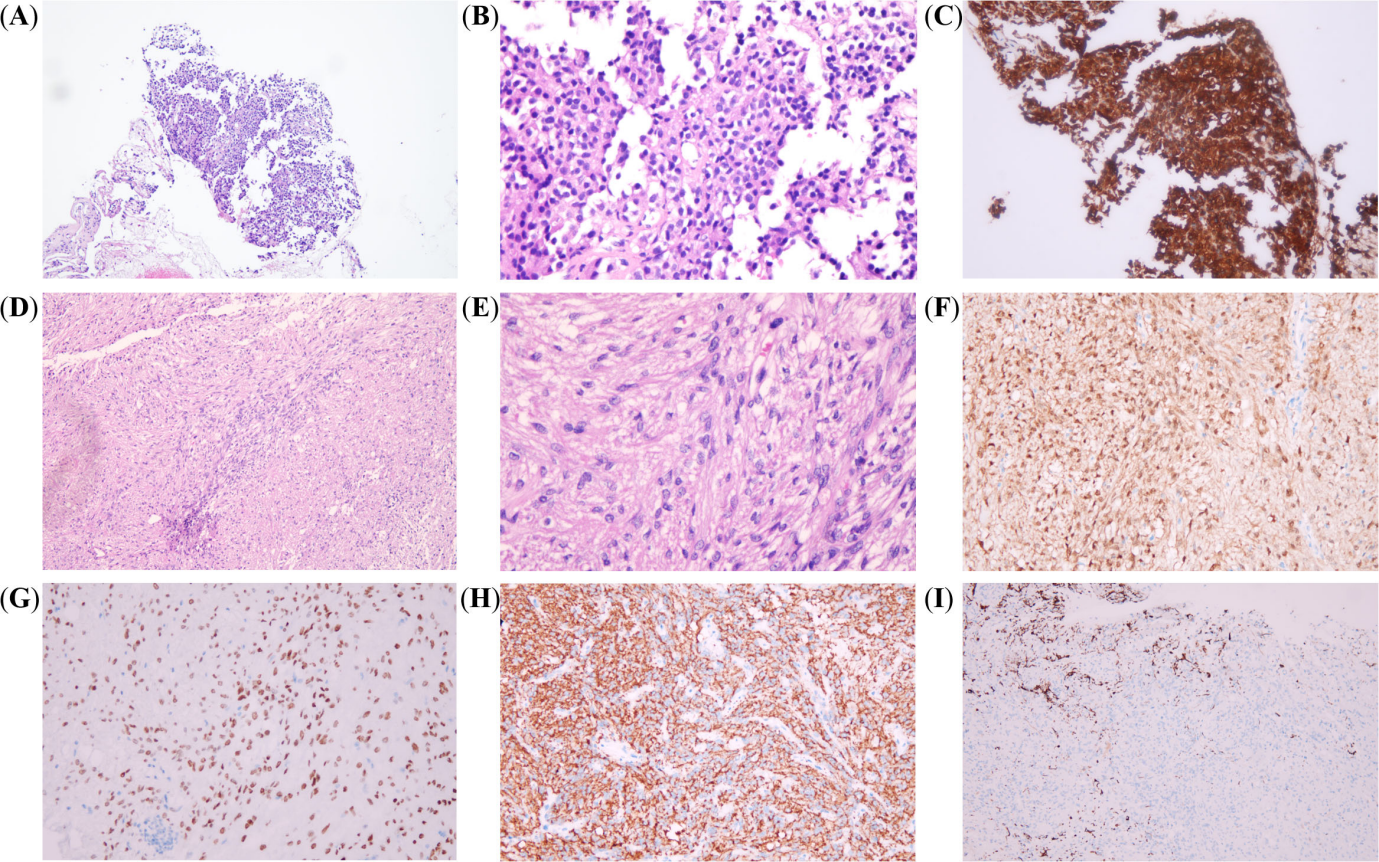
Immunohistochemical analysis. The two types of tumors were adjacent but not in direct contact. (**A** [×100] and **B** [×400]) Basophilic cells with granular cytoplasm and round nuclei; maximum diameter of the tumor was less than 2 mm. (**C** [×400]) IHC staining for ACTH was positive. (**D** [×100] and **E** [×400]) Spindle cells with interlacing fascicles and poorly defined lobules; cytoplasm was eosinophilic, and nuclei were oval, spindle, and scatteredly elongated. (**F** [×200], **G** [×200], **H** [×200], and **I** [×200]) Positive IHC staining for S100, TTF-1 and Syn, and scatteredly positive for GFAP.

Endoscopic sinus surgery was performed to remove the mass in the right paranasal sinus, and *Aspergillus* was identified in the mass located in the right sphenoid sinus by immunohistochemistry. Because of concerns about a potential intracranial fungal infection following surgery, the patient was advised to undergo treatment with voriconazole 200 mg twice a day prior to undergoing surgery for Cushing’s disease. Three months later, she received another nasal endoscope, which showed clear maxillary and sphenoid sinuses. CT scan of the nose found no maxillary sinusitis and remarkable improved sphenoiditis. Consequently, the patient received a transsphenoidal surgery to remove the mass in the pituitary gland. During the surgery, a single, highly vascularized mass, measuring 5×6×4 mm, was identified in the sellar region. It compressed the normal pituitary tissue and invaded the dura in the floor of sellar turcica. The mass was completely removed. Hematoxylin–eosin (HE) staining of the excised tissue revealed the tumor, and immunohistochemical analysis confirmed the presence of two distinct types of tumors, which were adjacent but not in direct contact, with clear margins between them (**Figure 2**). One was a very small corticotroph adenoma, measuring less than 2 mm in diameter, which was positive for Syn, CK8/18, T-pit, and ACTH, but negative for TTF-1, GFAP, and other pituitary hormones. Ki-67 was less than 3%. The other tumor consisted of spindle-to-epithelioid tumor cells with variable eosinophilic cytoplasm, arising from poorly defined lobules and interlacing fascicles, as seen on HE staining. Immunohistochemistry showed positivity for Syn, S100, and TTF-1; scatteredly positive for GFAP; and negativity for all anterior pituitary hormones. Ki-67 was also less than 3%. Therefore, both corticotroph adenoma from anterior pituitary and oncocytic pituicytoma from posterior pituitary were simultaneously identified. The patient’s serum cortisol the morning after surgery was 51 nmol/L, indicating complete remission. No recurrence was observed based on clinical examination and MRI during the 32-month follow-up period.

## 3 Review of the literature

We conducted a search on PubMed from the inception until June 2024, using search terms “Cushing’s disease” or “hypercortisolism” in combination with “posterior pituitary tumors”, “pituicytoma”, “granular cell tumor”, “spindle cell oncocytoma”, or “sellar ependymoma”. No restrictions were placed on language or publication type. The authors added gray literature with their expertise. We also reviewed the reference lists of relevant narrative reviews related to the topics identified in our search.

Based on the search strategy described above, only 23 cases of PPT coexisting with hypercortisolism, including the present case, have been reported to date (**Table 1**). Among these, 19 (82.6%) were identified as pituicytomas, 2 were identified as granular pituicytomas (GCTs), and 2 were identified as oncocytic pituicytomas (SCOs). To date, there have been no reported cases of Cushing’s disease associated with ependymal pituicytomas (sellar ependymomas). It is important to note that not all of the original publications for the 23 patients listed in **Table 1** provided a detailed account of the preoperative endocrine evaluation. For example, in the cases of patient #8, #17, and #18, the original reports mentioned a diagnosis of Cushing’s disease but did not include detailed descriptions of the preoperative endocrine workup, which is essential for confirming the diagnosis. Similarly, for patient #2, a definitive diagnosis of Cushing’s disease was not established before surgery. However, postoperative findings, including a significant reduction in serum cortisol levels and resolution of Cushing’s syndrome, strongly suggest a diagnosis of Cushing’s disease. Despite the incomplete documentation of endocrine assessments in some cases, all 23 patients were ultimately diagnosed with Cushing’s disease and underwent surgical treatment.

**Table 1:** Review of cases in the literature of coexistent pituicytoma and Cushing’s syndrome.

	Author	Age/Sex	Clinical features	Preoperative endocrine workup	Tumor size (mm)	MRI	IPSS	Surgical approach	Finding during the surgery	Preoperative diagnosis	Pathological finding of PPT	Pathological finding of CA or CH	Prognosis
1	Schmalisch, 2012 (6)	48/M	CS, HTA, DM, and pathological fractures	Elevated 24-h UFC; failure of low-dose DST, and successive high-dose DST	4	No pituitary tumor could be clearly identified; two suspicious areas were found	Performed before the 2nd surgery; no clear side preference	TS	Tumor was small and contiguous to the posterior pituitary gland	CD	Pituicytoma (positive IHC for S100, TTF-1, GFAP; negative IHC for Syn and EMA and pituitary hormones)	Not identified; Crooke’s hyalinization	No remission after 1st surgery. Hemihypophysectomy on the right side was performed, and remission was achieved.
2	Chakraborti, 2013 (7)	24/M	CS, HTA and pedal edema	Elevated ACTH and serum cortisol; loss of cortisol circadian rhythm	6×4	Hypointense on T1, hyperintense on T2 with peripheral rim enhancement. Located in sellar, compressed and expanded the pituitary gland anteriorly	NA	EETS	Mass was small, soft and suctionable, and surrounded by normal pituitary.	NA	Pituicytoma (positive IHC for S100, TTF-1, vimentin, GFAP, EMA; IHC for pituitary hormones NA)	Not identified	Remission
3	Ciccone, 2014 (8)	6/F	Growth failure, weight gain, premature pubarche, hypertrichosis	Elevated 24-h UFC; loss of cortisol circadian rhythm; failure of low-dose DST, and successive high-dose DST	Not indicated	Convex upper surface, focal T2-weighted hyperintensity in the middle region	Yes, midline lesion was suggested	TS	Small and soft	CD	Pituicytoma (IHC NA)	CA identified in the 2nd surgery (positive IHC for ACTH and GH); tumor size NA	Persistent hypercortisolism after 1st surgery; remission after 2nd surgery
4	Cambiaso, 2015 (9)	7/F	CS, precocious pubarche, reduced growth velocity, weight gain, and muscular weakness	Elevated 24-h UFC; loss of cortisol circadian rhythm; failure of low-dose DST, and successive high-dose DST	Not indicated	A small T2-weighted hyperintensive area on the mesial and upper portion; a small T1-weighted hypointense area in median and paramedian left side	Yes, midline lesion was suggested	MTS+EETS	No frank adenoma was found; a well-circumscribed area of pathologic tissue on the left side was removed	CD	Pituicytoma (positive IHC for S100, vimentin and GFAP; negative IHC for EMA and Syn; IHC for pituitary hormones NA)	CA identified in the 2nd surgery (positive IHC for ACTH, GH and PRL)	No remission after 1st and 2nd surgery. Bilateral laparoscopic adrenalectomy was performed.
5	Guo, 2016 (10)	46/F	CS and headache	Elevated ACTH, serum cortisol and 24-h UFC; failure of low-dose and 48-h 2 mg of DST	15×10×7	An abnormal enlarged parenchymal lesion in the left inferior pituitary gland; equal T1 and T2 signals, marked homogeneous enhancement; a thickened pituitary stalk	NA	MTS	Tumor was reddish, soft, and bled easily; within the sellar turcica	CD	Pituicytoma (positive IHC for S100, NF and GFAP; negative IHC for EMA, Syn and CD34; MIB-1 1%; IHC for pituitary hormones NA)	CH, positive IHC for ACTH	No remission, radiotherapy was performed.
6	Barresi, 2017 (11)	53/F	CS and asthenia	Loss of cortisol circadian rhythm; failure of low-dose DST, and successive high-dose DST	5×5×7	A small hyperintense lesion in the right side of the pars intermedia of the pituitary gland	Yes, right-sided dominance	EETS	Yellowish soft tissue	CD	Pituicytoma (positive IHC for S100, vimentin, TTF-1, EMA and GFAP; negative IHC for CgA, Syn, CK or pituitary hormones; Ki-67 less than 1%)	Not identified	Remission
7	Zhang, 2018 (12)	32/M	CS, polyuria, polydipsia, fatigue, obesity, and decreased libido	24-h UFC was not suppressed in 24-h low-dose and large-dose DST	Not indicated	The mass enhanced obviously and heterogeneously, pituitary stalk was slightly thick	Yes, left-sided dominance	TS	Hypovascular, gray-whitish soft tissue	CD	GCT (positive IHC for CgA, Syn, and GFAP; negative IHC for S100; Ki-67 5%)	Not identified	Remission
8	Lefevre, 2018 (13)	56/F	CS, HTA, IGT and depression	Not available	/	No abnormality.	Yes, right-sided dominance	TS	NA	CD	Pituicytoma (positive IHC for TTF1, IHC for other markers NA)	CA, positive IHC for ACTH	Remission
9	Chang, 2018 (14)	53/F	CS	Serum cortisol 44.4 μg/dL; failure of low-dose DST, and successive high-dose DST	5.7×5.8×4.5	Left pars intermedia	NA	TS	NA	CD	Pituicytoma (positive IHC for S100, Vimentin, TTF1, ADH, Syn, EMA, and BCL-2; negative IHC for GFAP, and CK)	Not identified	Remission
10	Chang, 2018 (14)	51/F	CS	Serum cortisol 30.2 μg/dL; failure of low-dose DST, and successive high-dose DST	6.5×6.5×7.6	Right paramedian pars distalis	NA	TS	NA	CD	Pituicytoma (positive IHC for S100, Vimentin, TTF1, ADH, Syn, EMA, and BCL-2; negative IHC for GFAP, and CK; IHC for pituitary hormones NA)	Not identified	No remission, radiotherapy was performed.
11	Chang, 2018 (14)	57/F	CS	Serum cortisol 22.5 μg/dL; failure of low-dose DST, and successive high-dose DST	5.1×2.2×3.3	Left paramedian sellar floor	NA	TS	NA	CD	Pituicytoma (positive IHC for S100, Vimentin, TTF1, ADH, Syn, and BCL-2; negative IHC for GFAP, EMA, and CK; negative IHC for pituitary hormones)	CA identified, positive IHC for ACTH and LH	Remission
12	Gezer, 2019 (15)	37/M	CS and blurry vision	Elevated 24-h UFC; loss of cortisol circadian rhythm; failure of 2-day DST, and successive high-dose DST	6×6.5	Lesion with isotense in T1 and T2 images, enhanced on the pituitary stalk	NA	EETS	NA	CD	Pituicytoma (positive IHC for S100 and TTF1; negative IHC for GFAP; Ki-67 1%; IHC for pituitary hormones NA)	Not identified	Remission
13	Li, 2019 (16)	32/F	CS	Elevated serum cortisol; failure of low-dose DST, and successive high-dose DST	7.5×5.7	A sellar mass with slightly hypointensity on T2-weighted images	NA	TS	NA	CD	Pituicytoma (positive IHC for S100, vimentin and TTF1; negative IHC for EMA, Syn, PAS and pituitary hormones; Ki-67 less than 1%)	Not identified	Remission
14	Guerrero-Pérez, 2019 (17)	62/F	CS	Elevated serum cortisol and 24-h UFC; failure of low-dose DST, and successive high-dose DST	/	No abnormality.	Yes, left-sided dominance	TS	Small, firm, and fusiform tumor behind the pituitary gland	CD	GCT (positive IHC for S100, vimentin and TTF1; IHC for pituitary hormones NA)	CH, positive IHC for ACTH	No remission, ketoconazole was prescribed.
15	Feng, 2020 (18)	29/F	CS	Elevated ACTH, serum cortisol and 24-h UFC; failure of low-dose DST and successive high-dose DST	4	Hypointense on T1, hyperintense on T2; contrast enhancement was intense and homogeneous.	NA	TS	Soft and vascular; demarcation was not very clear	CD	Pituicytoma (positive IHC for S100, TTF-1, and GFAP; negative IHC for EMA)	Not identified	Remission
16	del Pont, 2020 (19)	33/F	CS	Elevated serum cortisol and 24-h UFC; loss of cortisol circadian rhythm; failure of low-dose DST	<10	A microadenoma lesion in the right side of the gland.	NA	TS	NA	CD	Pituicytoma (positive IHC for S100, vimentin and TTF1; negative IHC for Syn; IHC for pituitary hormones NA)	Not identified	Remission
17	Rumeh, 2020 (20)	47/F	CS	Elevated serum cortisol and ACTH	5.5	A small non-enhancing nodule in left side of adenohypophysis	NA	TS	NA	CD	Pituicytoma (positive IHC for S100, TTF1 and GFAP; IHC for pituitary hormones NA)	Not identified	NA
18	Saeger, 2021 (5)	47/F	CS	Not available	/	No abnormality.	NA	TS	A tumor measuring 1 mm and adjacent grayish soft tissue	CD	Oncocytic pituicytoma (SCO) (positive IHC for S100, TTF1 and GFAP; negative IHC for pituitary hormones; Ki-67 3%)	CA, Crooke cell tumor, positive IHC for ACTH	NA
19	Xiao, 2022 (21)	51/F	CS, HTA, IGT, and hyperlipidemia	Elevated ACTH and 24-h UFC; loss of cortisol circadian rhythm; failure of low-dose DST, and successive high-dose DST; responsive to DDAVP stimulation test	9.7×4.0	A lesion at the left side of the pituitary gland, with a thickened pituitary stalk and the loss of T1 signal hyperintensity in the posterior pituitary gland	NA	TS	A soft white mass at the left side and a white-gray tough lesion in the posterior pituitary	CD	Pituicytoma (positive IHC for TTF1, GFAP, EMA and cytokeratin AE1/AE3; negative IHC for pituitary hormones)	CA, positive IHC for ACTH, T-pit; Ki-67 10%	Remission
20	Xiao, 2022 (21)	29/M	CS, HTA, renal calculus, rib fractures, and lumbar compressional fractures	Elevated ACTH and 24-h UFC; loss of cortisol circadian rhythm; failure of low-dose DST, and successive high-dose DST	3×4	A hypointense nodule at the left side of the pituitary	Yes; left-sided dominance	EETS	White-gray, soft, and poorly vascular lesion	CD	Pituicytoma (positive IHC for S100, TTF-1, Syn, and GFAP; negative IHC for SSTR-2 and AE1/AE3; IHC for pituitary hormones NA)	Not identified	Remission
21	Wee, 2023 (22)	71/F	CS, HTA, stable ischemic heart disease, hyperlipidemia, pre-diabetes, vertebral fracture	Elevated ACTH and 24-h UFC; failure of low-dose DST	6×4 (L); 10×6 (R)	Left: T2 hyper-intensity and early hyper-enhancement; Right: T2 iso-intensity and delayed enhancement	Yes; right-sided dominance	TS	A soft, pale, flaky lesion in the right side and firm, gritty tissue in the left side	CD	Pituicytoma (positive IHC for S100, TTF1 and GFAP; negative IHC for EMA and pituitary hormones)	CA, Crooke cells were seen, positive IHC for ACTH	Remission
22	Rubino, 2023 (23)	41/F	CS, HTA, DM, leg weakness, headaches, and visual difficulty	Elevated ACTH and serum cortisol; failure of low-dose DST	8×14×9 (L); 6×11×5 (M)	Left: T2WI hypointense, slightly T1WI hyperintense lesion in the anterior aspect; Midline: T2WI hyperintense, slightly T1WI hyperintense lesion in the posterior aspect	NA	EETS	Soft, not bleeding and easily dissectible	CD	Pituicytoma (positive IHC for TTF1 and B cell lymphoma 2; IHC for pituitary hormones NA)	CA, positive IHC for ACTH; negative IHC for p53; Ki-67 less than 1%	Remission
23	This case	41/F	CS, HTA, IGT, hyperinsulinemia and sphenoiditis	Elevated serum cortisol and 24-h UFC; loss of cortisol circadian rhythm; failure of low-dose DST, and successive high-dose DST; responsive to DDAVP stimulation test	6	A slightly enhanced nodule in the right lower lobe of the pituitary gland	NA	TS	A mass rich in blood supply with a size of 5×6×4 was found in the sellar region	CD	Oncocytic pituicytoma (positive IHC for S100, TTF1, Syn and GFAP; negative IHC for pituitary hormones; Ki-67 less than 3%)	CA, positive IHC for ACTH, T-pit, Syn, CK8/18	Remission

M, male; F, female; CA, corticotroph adenoma; CH, corticotroph hyperplasia; CS, Cushing syndrome; HTA, hypertension; DM, diabetes mellitus; IGT, impaired glucose tolerance; 24-h UFC, 24-h urinary free cortisol; ACTH, adrenocorticotropin; DST, dexamethasone suppression test; DDAVP, desmopressin acetate; CD, Cushing’s disease; IPSS, inferior petrosal sinus sampling; TS, transsphenoidal surgery; EETS, endoscopic endonasal transsphenoidal surgery; MTS, microscopic transsphenoidal surgery; IHC, immunohistochemistry; GCT, granular cell tumor; SCO, spindle cell oncocytoma; NA, not available.

Pathological analysis revealed corticotroph adenomas in nine cases and corticotroph hyperplasia in two cases. Among the nine patients with corticotroph adenomas, two required a second surgery before tumor identification. In 12 cases, neither corticotroph adenomas nor hyperplasia was identified. Notably, nine patients achieved remission of Cushing’s syndrome after surgery, although prognosis data were unavailable for one patient. Pathological examination of all 23 PPTs showed no evidence of pituitary hormone secretion, consistent with previous reports that PPTs lack ACTH secretion capability.

## 4 Discussion

Approximately 20 cases of PPTs have been reported following surgery for Cushing’s disease, as determined by pathological examination. The majority of these cases were diagnosed as pituicytomas, with only one case identified as an oncocytic pituicytoma (previously named SCO) (5). It is important to note that although most subjects in these studies achieved remission of Cushing’s syndrome after surgery, corticotroph adenoma or hyperplasia was identified in a fraction of cases by pathological examination. As a result, some researchers have hypothesized that PPT could produce some substances that might stimulate anterior pituitary hormone hypersecretion (18). In this study, we presented the second case of Cushing’s disease coexisting with oncocytic pituicytoma. Pathological examination demonstrated a corticotroph adenoma with a diameter of less than 2 mm, alongside a larger oncocytic pituicytoma.

PPTs are exceedingly rare tumors. While functional pituitary adenomas, such as corticotroph and GH adenomas, are relatively more common than PPTs, they remain uncommon overall. In addition to hyperprolactinemia caused by mass effect, patients with PPT have been found to exhibit a relatively high prevalence of adenohypophyseal hyperfunction. A systematic review that summarized all published clinical cases with PPT indicated a prevalence of 5.6% (15/226), including 10 cases with hypercortisolism and 5 cases with acromegaly (3). The German Pituitary Tumors Registry identified 69 PPTs, among which four subjects had coexisting functional pituitary adenomas—two with prolactin-producing tumors, one with GH tumor, and another one with Crooke cell tumor (5). To our knowledge, only three cases of concomitant PPTs and non-functional anterior pituitary adenomas have been reported (5, 24). In other words, because both PPTs and anterior pituitary adenomas are rare, and based on published literature, several cases of coexisting PPT and functional pituitary adenoma have been documented. Some researchers believe that the association between PPTs and adenohypophyseal hyperfunction is not merely coincidental (4). Possible mechanisms have been proposed to explain this coexistence. For example, cells from PPTs might induce stimulation signals and promote cell proliferation in adjacent adenohypophyseal neurosecretory cells or regulate hormones in the hypothalamus (18). However, it may also be due to the reason that adenohypophyseal hyperfunction makes individuals with PPTs more likely to receive surgery at an early stage. This could be supported by the evidence showing that the mean tumor size of sporadic PPT reported so far is much larger than that of PPTs with adenohypophyseal hyperfunction (4). The actual prevalence of PPTs may not be as low as it appears. Since almost all the subjects with PPTs were diagnosed incidentally after surgery due to mass effect, there may be additional undiagnosed cases of PPTs, which were not identified because of their benign and slow-growing nature.

In our literature review, only one subject (patient #18) has been reported where corticotroph adenoma coexisted with oncocytic pituicytoma (SCO). Specifically, the patient was considered to have Crooke cell adenoma, which is a very rare type of corticotroph adenoma characterized by Crooke hyaline changes in tumor cells. Clinical features of this case were not described in detail. Therefore, our study is the first to present detailed clinical data of a patient with corticotroph adenoma associated with oncocytic pituicytoma (SCO). Comprehensive endocrine evaluations confirmed the diagnosis of Cushing’s disease before surgery. MRI before surgery identified a mass in the sellar region, which was likely an image of the oncocytic pituicytoma based on the tumor’s size. The corticotroph adenoma was likely too small to be visualized by the MRI. We assume that the corticotroph adenoma was located near the oncocytic pituicytoma; thus, when the oncocytic pituicytoma was resected, the corticotroph adenoma was also removed simultaneously. Preoperative MRI scans identified two pituitary lesions in only two cases (patient #21 and #22). While the median tumor size of sporadic PPTs reported by far is 22.0 mm (3), the largest mass among the 23 cases was 15 mm in diameter. MRI did not detect any lesions in three patients (patient #3, #4, and #22), likely because hypercortisolism-related symptoms led to the early discovery of PPTs before the tumors grew large enough to cause mass effects.

Both patient #18 and our case of oncocytic pituicytoma (SCO) were positive for S100, TTF1, and GFAP, and negative for pituitary hormones by immunohistochemistry (IHC). Ki-67 was 3% in one patient and less than 3% in the other. Notably, the histomorphology of the PPTs among some of the 23 cases appears more typical for a oncocytic pituicytoma (SCO) than for a pituicytoma, according to both our research team and other investigators. Historically, the term pituicytoma has been used to describe various tumors with spindle cells in the sellar region (25). As a result, pituicytoma diagnosed in previous studies might not meet the traditional pituicytoma based on the 2022 WHO criteria. Additionally, the differential diagnosis between SCO and pituicytoma can sometimes be challenging, as they may present a wide range of overlapping histological features (26). Some researchers have even considered SCO to be a morphological variant of pituicytoma (26, 27). Therefore, in 2022, the 5th Edition of the WHO Classification of Endocrine and Neuroendocrine Tumors proposed that SCO, GCT, and sellar ependymoma should be classified as subtypes of pituicytoma (1). In other words, there might be more than two cases of Cushing’s syndrome associated with oncocytic pituicytoma.

The immunoprofile of oncocytic pituicytomas typically includes markers such as vimentin, S-100 protein, EMA, TTF-1, and somatostatin receptors (28). GFAP expression is less commonly observed and is typically limited to a small subset of cells. In the present case, the immunohistochemical findings were positive for Syn, S-100, and TTF-1, with scattered GFAP positivity and negativity for anterior pituitary hormones. These results are consistent with a diagnosis of oncocytic pituicytoma. However, distinguishing between PPTs based solely on histological and immunohistochemical features can be challenging due to overlapping characteristics. Recent genetic and epigenetic studies have identified subtle DNA methylation variations, mutation patterns, and differing clinical outcomes among PPTs. These findings suggest that molecular diagnostics could provide valuable insights for tumor subclassification (29). Nevertheless, the routine clinical application of these molecular techniques has not been fully explored and requires further research to determine their role in the classification and management of posterior pituitary neoplasms.

Our study highlights the challenges in diagnosing Cushing’s disease. Inferior petrosal sinus sampling (IPSS), considered the gold standard for confirming the source of ACTH secretion, remains underutilized due to its technical complexity, procedural risks, and the requirement for specialized expertise (30, 31). In our study, only 9 of 23 patients underwent IPSS, reflecting these limitations. According to the 2021 Pituitary Society guidelines, the diagnostic approach should prioritize non-invasive tests, such as late-night salivary cortisol, DSTs, and 24-h urinary free cortisol (32). These tests provide essential information, while pituitary MRI may be instrumental in distinguishing between ectopic and pituitary ACTH sources. IPSS is essential when biochemical and imaging results are inconclusive but may not be necessary if a pituitary tumor ≥10 mm or <6 mm is identified along with supportive biochemical evidence. In contrast, for tumors measuring between 6 and 9 mm, expert opinions vary; nevertheless, the majority recommend using IPSS for diagnosis in such cases (32). However, practical challenges persist, as not all centers have the resources or expertise to perform all recommended tests, particularly IPSS, leading to potential diagnostic delays. This underscores the importance of a multidisciplinary approach to ensure accurate diagnosis and optimal management of Cushing’s disease, taking into account the available resources.

In our case, the PPT was located in the lower anterior part of the pituitary, which could easily be mistaken for a pituitary adenoma (**Figure 1**). According to previous literature, pituicytomas (PPTs) are more commonly found in the suprasellar region (33–35). Salge-Arrieta et al. conducted a systematic analysis of imaging findings in 104 cases of pituicytoma and reported that PPTs were purely suprasellar in 22.1%, purely intrasellar in 41.3%, mixed sellar/suprasellar in 30.8%, and invaded the cavernous sinuses in 5.8% (33). Therefore, PPTs can originate anywhere along the hypophyseal–infundibulum–median eminence axis (33). In addition, the clinical presentation of PPTs is influenced primarily by tumor size and location. Among the reported cases, the most common presenting symptom was visual impairment (48.8%), followed by hypopituitarism (31.5%) and headache (25.6%) (35). Patients with suprasellar PPTs frequently experience isolated visual symptoms due to optic nerve compression, whereas intrasellar tumors more often result in headache and hypopituitarism due to compression of the pituitary gland or adjacent structures.

Recent studies highlight the role of frailty in outcomes for various conditions, including Cushing’s disease (36). Mild frailty, as assessed by the 11-factor modified frailty index, has been shown to predict surgical outcomes and may assist in preoperative risk stratification (36). In our study, we aimed to assess frailty in all the cases included in the review; however, variability in the reported data limited our ability to apply standardized scoring. Future research will incorporate frailty assessments to provide more evidence on its impact in Cushing’s disease.

In summary, our study highlights the rare coexistence of Cushing’s disease and oncocytic pituicytoma, a condition reported in only one other case. Hypercortisolism associated with PPT presents significant diagnostic challenges, as corticotroph adenomas or corticotroph hyperplasia are often undetected both preoperatively and, in some cases, postoperatively due to their small size. This emphasizes the need for improved diagnostic tools and surgical techniques to identify these lesions more reliably. Future research should focus on advanced imaging modalities with enhanced sensitivity and innovative intraoperative methods, such as real-time fluorescence-guided surgery and advanced pathological staining. Additionally, larger, multicenter studies are essential to uncover the mechanisms underlying the coexistence of these conditions and to inform the development of optimal diagnostic and therapeutic strategies.
